# The next frontier in healthcare simulation is human-AI teaming

**DOI:** 10.1186/s41077-026-00479-y

**Published:** 2026-10-09

**Authors:** Sonia Jawaid Shaikh, Walter Eppich

**Affiliations:** 1 School of Culture and Communication, The University of Melbourne, Melbourne, Victoria Australia; 2 Department of Medical Education and Collaborative Practice Centre, Faculty of Medicine, Dentistry and Health Sciences, The University of Melbourne, Melbourne, Victoria Australia

## Abstract

**Background:**

Across health professions, AI agents are moving out of the background and into clinical teams as collaborative entities. Yet healthcare simulation continues to conceptualise AI as a tool that serves the scenario rather than one that inhabits it. This framing is no longer adequate. The clinical environments we are preparing practitioners for demand that professionals learn to team with AI and not merely use it.

**Main body:**

We contend that human-AI teaming (HAIT) is the next frontier for healthcare simulation education and research. The addition of AI agents to human teams fundamentally alters how teams communicate, work together, and accomplish goals and therefore, by extension, how we must design simulation systems for education and research. We identify five dimensions of teamwork that AI is reshaping and discuss what each means for simulation practice: communication and information exchange, shared mental models, trust calibration, team reflexivity, and team performance. We then present five imperatives that healthcare simulation must address to meet the challenges and opportunities HAIT introduces: fidelity, assessment, ethics, governance, and medico-legal framing, curriculum and faculty development, and transfer of learning. Together, these ten domains constitute a research and education agenda for a field built on the science and art of preparation.

**Conclusion:**

HAIT is an emerging clinical reality and the next frontier for healthcare simulation. We call on the simulation community to treat it as a first-order priority and to focus on developing fidelity standards for AI agents in simulation, validating human-AI teaming assessment tools, establishing ethical frameworks for AI-inclusive scenario design, investing in curriculum and faculty development, and committing to the research needed to establish whether HAIT-based simulation training translates into improved performance in AI-augmented clinical practice.

## Introduction

Year 2030. A team comprising neurosurgeons, anaesthetists, nurses, and surgical technicians works collaboratively to perform a complex neurosurgical procedure. An intelligent decision-support system is present, integrating findings from sophisticated neuroimaging to assist the team. During the procedure, the intelligent system suggests a course of action with which not all team members agree. How should the team engage with the assistant, evaluate its recommendation, and decide on the best course of action?

The healthcare simulation field deals with anticipation, prediction, and preparation. Simulation innovators and practitioners look ahead and ask: how do we prepare people for future clinical practice before it arrives? This forward-looking approach allows us both to train professionals and teams and to develop implementable protocols and procedures to benefit patients. However, we now find ourselves at a unique moment in our history. Across health professions, human teams are undergoing rapid evolution as AI agents move out of the background and start to integrate within them. From intensive care units to specialists’ offices, health professionals are increasingly *collaborating* with AI systems that flag deteriorating patients, recommend drug dosing, interpret imaging, and present diagnostic alternatives in *real time* [1]. With such technological advancement underway, we foresee human-AI teaming (HAIT) to be the emerging future of medicine which requires dynamic, adaptive, and reflexive responses. New perspectives on AI in simulation have developed useful conceptualisations on how AI can augment simulation specifically its education technology function [1]. Yet, in much of healthcare simulation, AI is still conceptualized as a tool that serves the scenario rather than one that inhabits it [2]. It is a background feature which generates scenarios and analyses performance, but it does not join the team. This framing was adequate when AI was genuinely a background technology with limited scope. However, recent developments will outpace our theoretical approaches and training protocols, rendering this framing inadequate. Our future healthcare work environments demand that we prepare health professionals for more than the ability to *use* AI, but the capacity to *team* with it where it will hold roles, make serious recommendations, and influence relational team dynamics.

We contend that HAIT is the next frontier in healthcare simulation. Schmutz et al. [3] defined human-AI teams as a type of team in which at least one human works together with at least one autonomous agent possessing a partial or high degree of self-governance over decision-making, adaptability, or communication, toward a common goal. What distinguishes HAIT from conventional human-computer interaction is precisely this collaborative attribute: AI agents are not passive instruments but active participants capable of generating recommendations, flagging disagreement, and adapting their behaviour based on team decisions. The addition of AI agents as members of healthcare teams fundamentally alters how teams think, communicate, work together, and accomplish goals and by extension, the ways we must design simulation systems for education and research. We identify five dimensions of teamwork that are qualitatively different in human-AI configurations compared to human-only teams and discuss what each means for simulation practice. These five dimensions do not simply describe how human-AI teams differ from human-only configuration. They reveal where our current simulation infrastructure falls short. Each one surfaces a gap that the field has not yet resolved. To address these changes, we also present five methodological, ethical, and educational imperatives for healthcare simulation that must be tackled to meet the demands of the emerging future in HAIT.

### Five changing dimensions of teamwork and implications for healthcare simulation

**Fig. 1:**
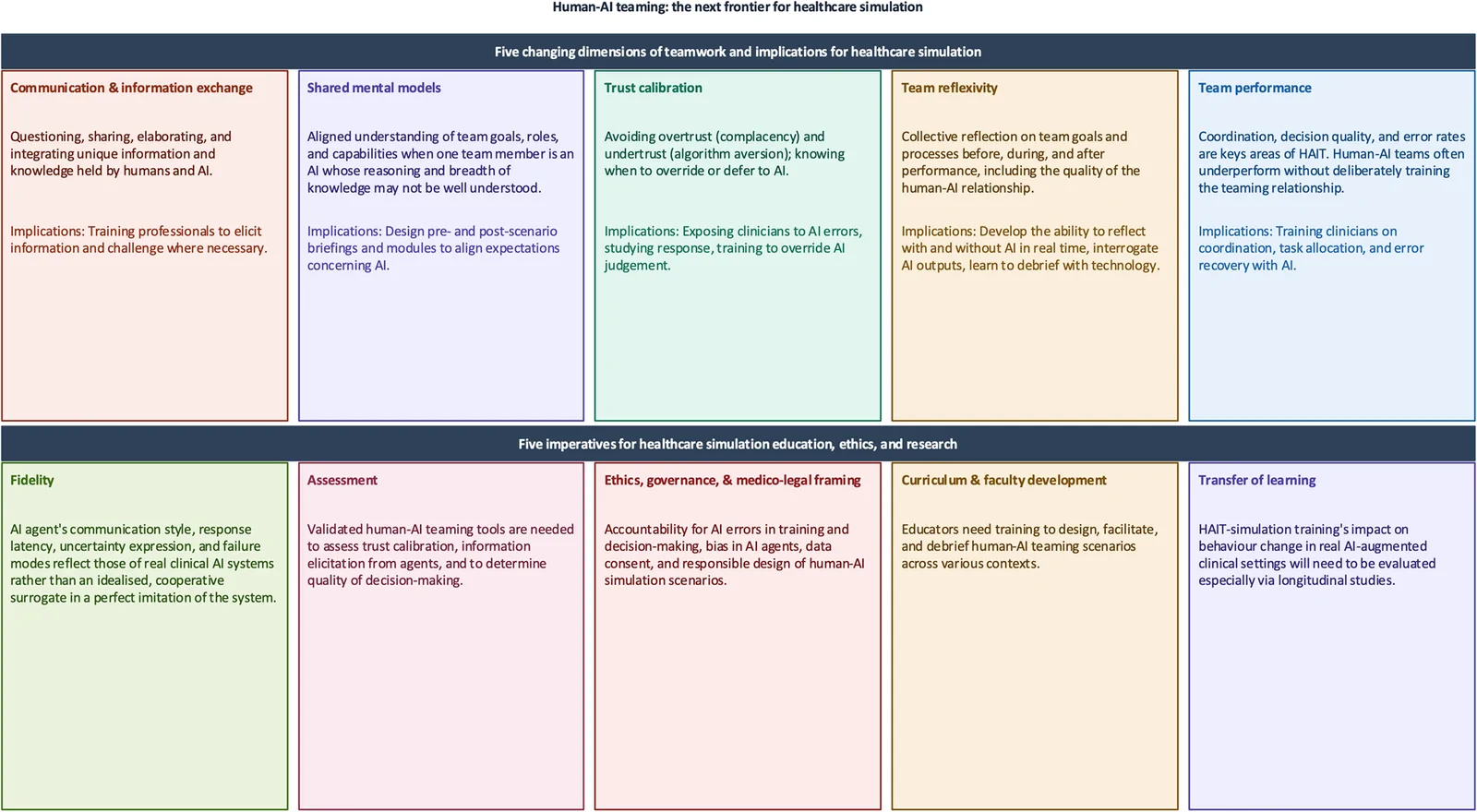
Human-AI teaming: dimensions of teamwork, implications, and imperatives for healthcare simulation

The science of HAIT is in its infancy. Yet, we can already identify five key dimensions on teamwork from team science where AI is already producing deep changes. These dimensions of teamwork carry direct implications for simulation design and research (Fig. 1).

#### Communication and information exchange

The type, quality, and extent of information exchange such as inquiring, probing, challenging, and discarding information is fundamentally different between human-only and human-AI teams. For example, research from human-AI teaming shows that human members of human-AI teams revert to minimal, task-focused exchanges, forgoing the rich elaboration of information that characterises effective human teams [4]. Although research on communication and information exchange in human health care teams is generally scarce, this finding as applied to health care simulation means we must train clinicians not only to communicate with AI agents using directed, closed-loop language, but also to actively elicit unique AI-generated information. The data AI holds may never surface if humans do not know to ask for it, to evaluate it, and to act on it.

#### Shared mental models

Aligned understanding of team goals, roles, and capabilities becomes harder to achieve when one team member is an AI whose reasoning and breadth of knowledge may not be well understood. This challenge extends to how teams develop transactive memory systems (TMS) in which knowledge and memory about who does what is cognitively distributed amongst team members [5]. In HAIT, TMS must extend to include an accurate understanding of what the AI knows and when its outputs can be trusted. In simulation, this translates to a need for pre-scenario briefings that establish AI role, scope, and known limitations explicitly. Scenario designs, including debriefing practices, should explore and shift misaligned expectations rather than assuming they will resolve on their own.

#### Trust calibration

Working alongside AI requires clinicians to develop well-calibrated trust which neither defers to AI recommendations uncritically nor dismisses them without due consideration. Three failure modes concerning trust that are particularly relevant in clinical human-AI teams are: overtrust, in which clinicians accept AI advice without adequate scrutiny (misuse or complacency); overreliance, in which AI input is sought or followed even when it adds no value; and undertrust, in which clinicians reject AI advice even when it is clinically useful (disuse) [6–8]. Calibration of trust in technology and its impact on trust amongst human teammates will be a critical prerequisite for the successful integration of AI within teams. Simulation offers a safe learning environment in which we can deliberately expose clinicians to AI error, study how trust shifts in response, and train them to override AI recommendations without harm to patients.

#### Team reflexivity (TR)

Schmutz and Eppich conceptualise TR as a team’s capacity to reflect collectively on its goals, strategies, and processes before, during, and after performance and to adapt accordingly [9]. In human-AI teams, this capacity has added complexity: the team must reflect not only on its own functioning but on the quality of its relationship with the AI. These teams will need to develop the ability to communicate clearly, interrogate AI’s outputs appropriately, and determine if and how the AI’s contributions were integrated or ignored. Simulation, with its structured debriefing tradition, is the natural venue for developing this form of reflexivity.

#### Team performance

In human-AI configurations, how the team performs does not follow automatically from the performance of individual team members. Research shows that the value of human-AI combinations depends highly on task type and design: human teams tend to underperform on decision-making tasks compared to creative tasks with outcomes moderated by team structure [10]. One driver of this behavior is that human members revert to minimal and shallow exchanges with AI agents rather than richer coordination patterns observed in human-only teams [4] Critically, this means that performance outcomes are sensitive to task design and teaming conditions, which is precisely the domain simulation is positioned to address. The goal of simulation is not merely to expose clinicians to AI, but to train specific behaviours such as coordination, task allocation, error recovery, which allow human-AI teams to perform at levels that neither could achieve alone. Simulation provides controlled environments in which these behaviours can be systematically trained, varied and measured against defined performance outcomes.

### Five imperatives for healthcare simulation education, ethics, and research

To truly prepare ourselves for a HAIT future in healthcare simulation, we must address five educational, ethical, and research imperatives that will impact the ways we work and train professionals. We discuss these below.

#### Fidelity

The idea of fidelity must be reconceptualised for the HAIT context. We see a unique opportunity in the extent to which the psychology, appearance, and behaviour of AI and interaction with it not only reflect in the simulated system but also extend to research, education and performance outcomes [11]. Accomplishing this will require that the AI agent’s communication style, response latency, uncertainty expression, and failure modes reflect those of real clinical AI systems rather than an idealised, cooperative surrogate in a perfect imitation of the system. Without reevaluating notion of fidelity in its educational, psychological, and behavioral scope, AI-relevant simulation exercises will fail to live up to their potential.

#### Assessment

The non-technical skills tools that simulation has validated over two decades (e.g. ANTS, OTAS, TEAM) were designed to assess human-human interaction [12–14]. These tools don’t capture trust calibration, information elicitation from an AI agent, or the quality of override decisions. New validated instruments, grounded in the five dimensions described above, are needed before the field can claim to measure HAIT competency with any rigour.

#### Ethics, governance, & medico-legal framing

The integration of AI teammates into simulation raises questions that existing ethical and governance frameworks were not designed to answer. We are primarily concerned with attribution of responsibility, burden of acknowledgement when making decisions, bias in AI agents, and quality of training datasets. Beyond individual scenarios, the broader embedding of AI into clinical workflows will require responsible design of human-AI simulation scenarios. Explicit ethical frameworks and institutional governance structures are needed before the field can responsibly scale HAIT-based simulation.

#### Curriculum and faculty development

Simulation educators are skilled in designing and debriefing human team scenarios. However, they are not yet trained to design scenarios in which AI holds a defined role, to facilitate debriefs that interrogate human-AI dynamics, or to identify moments where trust was miscalibrated or information exchange failed. Developing this skilled workforce requires concrete curricular innovation. Scenario design must incorporate AI archetypes (e.g. AI misuse or disuse during patient deterioration). Debriefing frameworks must be extended to enable exploration of human-AI dynamics (e.g. ask humans via structure prompts how they responded to incorrect AI). Faculty development programmes must equip educators with conceptual and practical tools to design and facilitate these scenarios. Curriculum development in simulation must treat human-AI teaming as a unique and attention-worth pedagogical domain.

#### Transfer of learning

For HAIT training, this gap between training and its transfer to clinical practice is particularly consequential: a clinician who performs well in a simulated human-AI team scenario must also be able to calibrate trust, elicit information, and reflect on AI-team dynamics when working alongside real AI systems in real clinical settings. Longitudinal studies that follow trainees from simulation into AI-augmented zclinical practice are urgently needed.

## Conclusions

The neurosurgical team in our opening vignette is a prediction of upcoming clinical reality [15] which simulation has not caught up with yet. AI agents are already in healthcare teams, already generating recommendations, and already demanding responses that require trust, judgment, and coordination from their human colleagues. The dimensions of teamwork we have described are being reshaped in real clinical settings today, and the educators, curricula, assessment tools, and fidelity standards needed to train for this reality do not yet exist. Simulation has always been the field that looks ahead and asks how to prepare practitioners for the environments they are about to enter and before those environments arrive. That question has never been more urgent, and the answer has never been less clear. The new frontier is here and healthcare simulation must meet it.

## Data Availability

Not applicable.
